# Mucins protect against *Streptococcus pneumoniae* virulence by suppressing pneumolysin expression

**DOI:** 10.1172/JCI182769

**Published:** 2024-08-22

**Authors:** Jade Bath, Elisabet Bjånes, Cengiz Goekeri, Jeff Hsiao, Deniz Uzun, Geraldine Nouailles, Victor Nizet, Katharina Ribbeck

**Affiliations:** 1 Department of Biological Engineering and; 2 Microbiology Graduate Program, Massachusetts Institute of Technology, Cambridge, Massachusetts, USA.; 3 Division of Host-Microbe Systems and Therapeutics, Department of Pediatrics, UCSD, La Jolla, California, USA.; 4 Department of Infectious Diseases, Respiratory Medicine and Critical Care, Charite - Universitätsmedizin Berlin, Berlin, Germany.; 5 Ragon Institute of MGH, MIT, and Harvard, Cambridge, Massachusetts, USA.; 6 Skaggs School of Pharmacy and Pharmaceutical Sciences, UCSD, La Jolla, California, USA.

**Keywords:** Infectious disease, Microbiology, Bacterial infections, Glycobiology

**To the Editor:***Streptococcus pneumoniae* (*Spn*) is a common member of the human nasopharyngeal microflora; yet, this same bacterium inflicts tissue damage and considerable mortality worldwide (1). Identifying host mechanisms that mediate the virulence of *Spn* will enable therapeutic development to mitigate *Spn* infections.

Upon mucosal colonization, *Spn* encounters mucins, the extensive glycoprotein polymers that are integral to host defense (Supplemental Figure 1A; supplemental material available online with this article; https://doi.org/10.1172/JCI182769DS1). Mucins form a robust barrier and mediate interactions with pathogenic microbes (2). Despite their central role in host-pathogen interactions, the extent to which mucins protect against *Spn*-mediated damage remains unclear. To address this gap, we utilized natively purified porcine gastric MUC5AC, a mucin source that replicates structural and functional attributes of human airway MUC5AC (3).

To investigate the potential protective effect of mucins, we grew *Spn* TIGR4, an invasive human disease isolate, in the presence of MUC5AC and exposed relevant host cells to *Spn* culture supernatant. We found that MUC5AC-treated *Spn* was less toxic to A549 lung cells and primary human neutrophils (Figure 1A). This protective effect extended to isolated mucin glycans and porcine intestinal MUC2, but not to a pool of monosaccharides that comprise mucin glycans, or carboxy methylcellulose (CMC), a control gel-forming polymer (Figure 1, B and C and Supplemental Figure 1, B and C). These findings highlight a specific role of mucins and mucin glycans in reducing *Spn* cytotoxicity and open questions as to how mucins attenuate *Spn*.

To assess whether mucins impact virulence factor expression in *Spn*, we exposed *Spn* TIGR4 to MUC5AC or mucin glycans and measured gene expression through RNA-Seq. We found that MUC5AC and mucin glycans induced widespread gene expression changes (Figure 1, D and E). Strikingly, MUC5AC and glycans potently downregulated pneumolysin (*ply*), a key toxin and virulence factor implicated in tissue damage, transmission enhancement, and inflammatory responses (4, 5). Beyond *ply* regulation, mucins downregulated virulence genes including the *blp* bacteriocins and *rlrA* pilus, while upregulating galactose metabolism genes. Quantitative reverse-transcription PCR (RT-qPCR) analysis of *ply* expression indicated that MUC5AC, along with mucins from other mucosal surfaces, MUC2, and MUC5B (human salivary mucin) reduced *ply* expression despite their different structures and glycan profiles, indicating a shared function. This effect was specific to mucins and mucin glycans, as CMC and a monosaccharide pool failed to suppress *ply* expression (Figure 1F). The downregulation of *ply* was consistent across different *Spn* serotypes, carbon sources, growth stages, and after short exposures to mucin (Figure 1G and Supplemental Figure 2, A–D). Western blot and hemolysis assay confirmed a decrease in active PLY protein after mucin exposure (Supplemental Figure 2, E and F). Notably, the decrease in PLY did not correlate with changes in bacterial growth, and *Spn* cannot utilize mucin or mucin glycans as a carbon source (Supplemental Figure 2, G–I). *Ply* regulation is not well understood, and disruption of putative regulators did not dampen the effects of mucin on *ply* expression, suggesting mucin acts through an uncharacterized mechanism (Supplemental Figure 3, A–C).

The reduced PLY expression is intriguing, considering its role in modulating the immune response. We confirmed that mucins protect neutrophil survival after exposure to live *Spn* (Supplemental Figure 4A); this protection was PLY-dependent, as the TIGR4Δ*ply* mutant, which does not express PLY, exhibited reduced cytotoxicity. To investigate the impact of mucin-PLY regulation on neutrophil function, we examined neutrophil activation and cytokine production. We measured the release of proinflammatory cytokine IL-1β, which is stimulated by PLY, and myeloperoxidase (MPO), a neutrophil activation marker. We observed reduced IL-1β (Figure 1H) and MPO (Supplemental Figure 4B) release when neutrophils were cocultured with mucin-treated *Spn*, approaching levels observed for TIGR4Δ*ply*.

The reduced neutrophil activation could suggest cellular inactivity, potentially compromising neutrophil phagocytosis. To address this, we examined whether mucin-treated *Spn* impacts neutrophil microbicidal function by measuring bacterial engulfment and killing. We cocultured GFP-tagged *Spn* with neutrophils and used flow cytometry and a gentamicin protection assay to assess phagocytosis and bacterial killing, respectively. We found that neutrophil phagocytosis and killing persisted in the presence of mucins (Figure 1I and Supplemental Figure 4, C and D), suggesting that mucins do not impair neutrophil function. The TIGR4Δ*ply* mutant exhibited dramatically reduced phagocytosis and killing, underscoring PLY’s role in neutrophil activation. This nuanced PLY regulation could reflect a balanced host-pathogen interaction, wherein host defenses eradicate pathogens without overactivation, leading to excessive inflammation.

Finally, to assess whether mucin or glycan exposure is sufficient to reduce virulence in vivo, we infected mice intratracheally with mucin- or glycan-treated *Spn*, administering an additional mucin or glycan treatment after 8 hours. After 24 hours, we analyzed recovered bacteria from the lung and blood and observed that mucin treatment significantly reduced lung bacterial levels (Figure 1, J and K). This mucin-*Spn* interplay highlights the key role of mucins in host defense and opens avenues for novel therapeutic interventions to diminish the virulence of *Spn*.

## Supplementary Material

Supplemental data

Unedited blot and gel images

Supporting data values

## Figures and Tables

**Figure 1:**
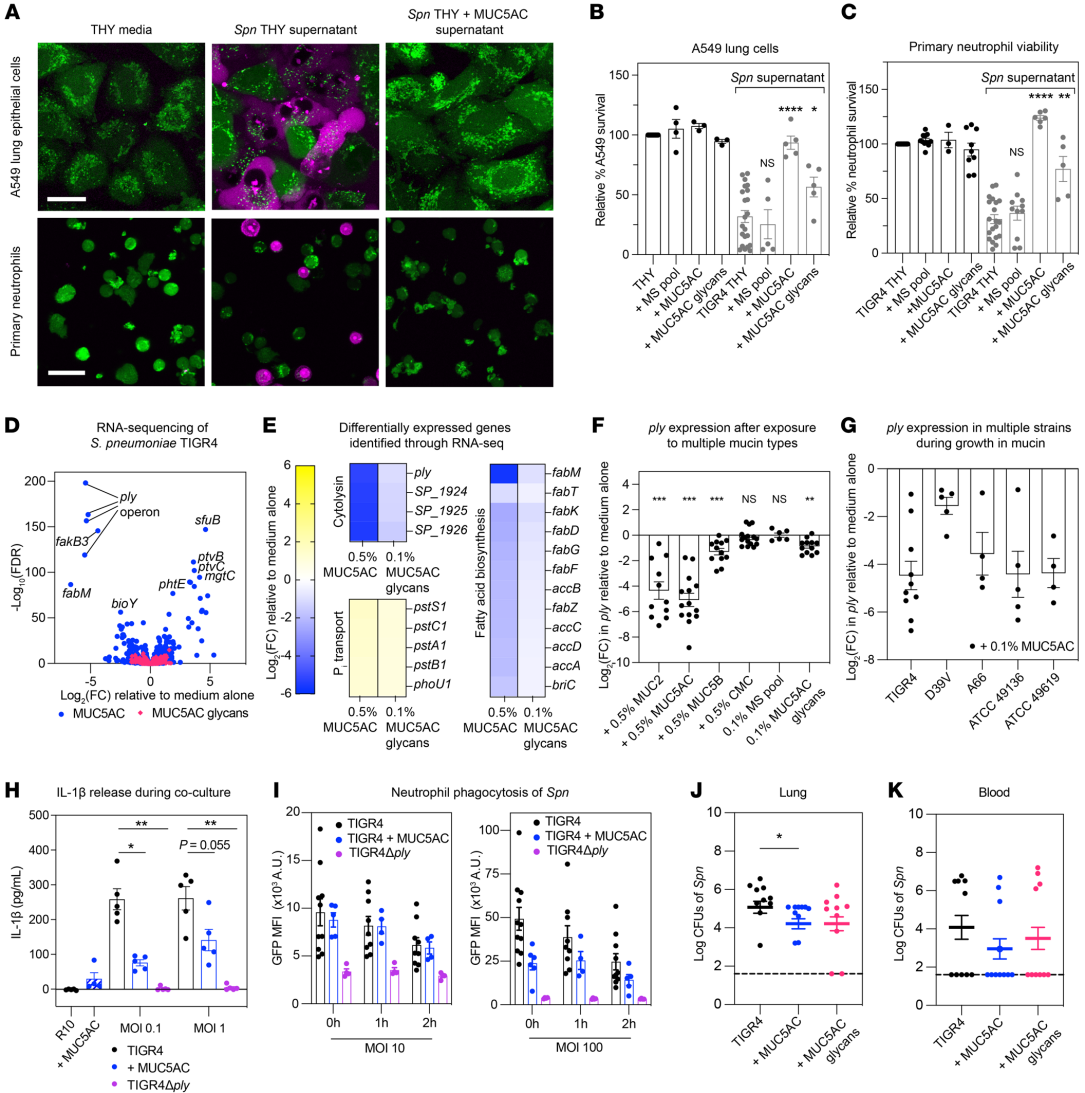
Mucins temper PLY expression to protect host cells, modulate immune responses, and attenuate infection. (**A**) Host cell survival after *Spn* supernatant exposure, shown by confocal microscopy using a LIVE (green)/DEAD (magenta) stain. Scale bar: 20 μm. (**B** and **C**) Host cell survival after mucin-, glycan-, or monosaccharide-treated (MS-treated) *Spn* supernatant exposure, measured by AlamarBlue. (**D** and **E**) MUC5AC and glycan exposure triggers transcriptional changes in *Spn* TIGR4 at 5 hours (average FC from three biological replicates). (**F** and **G**) RT-qPCR quantification of *ply* gene expression after 5 hour exposure to mucin isoforms (**F**) and in multiple *Spn* strains (**G**). (**H**) IL-1β release upon *Spn*-neutrophil interaction, measured by ELISA. (**I**) Neutrophil phagocytosis of GFP-tagged *Spn* TIGR4, with or without mucins, measured by flow cytometry. (**J** and **K**) Lung and blood bacterial burden in mice infected with mucin-treated *Spn*. *n* = 10/group. (**B**, **C**, **F**–**K**) Data represent mean ± SEM with biological replicates shown. (**B**, **C**, **H**) Mann-Whitney *U* test; (**F**) Wilcoxon test; (**J**–**K**) Kruskal-Wallis test with Dunn’s correction; *P < 0.05, **P < 0.01, ***P < 0.001, ****P < 0.0001.

## Version 1 08/22/2024

In-Press Preview

## Version 2 10/01/2024

Electronic publication
