# Supplementary material for: Mucins protect against Streptococcus pneumoniae virulence by suppressing pneumolysin expression
Source: J Clin Invest. 2024 Aug 22;134(19):e182769. doi: 10.1172/JCI182769 (PMC11444151; doi:10.1172/JCI182769)
Supplement: Supplemental data [file jci-134-182769-s037.pdf]

Supplemental Material

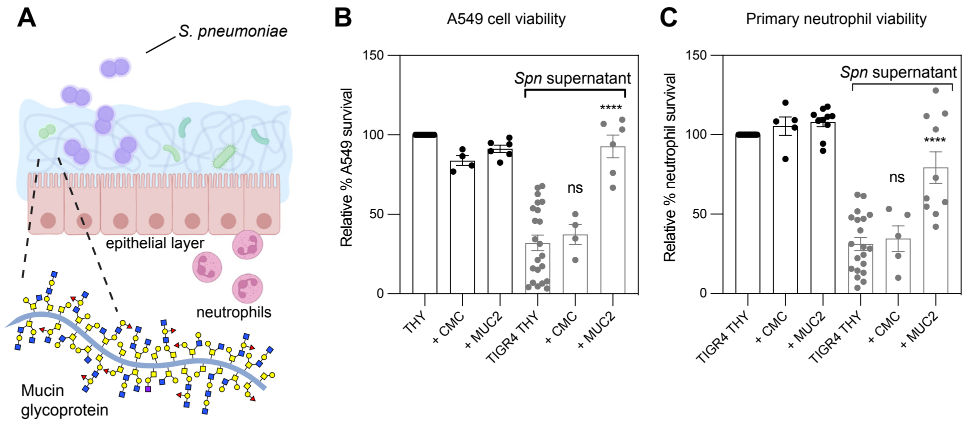

Figure S1. Mucins protect host cells independently of gel-forming properties. (A) The mucus barrier coats epithelia and creates a defensive habitat that *Spn* must navigate. **(B-C) Lung epithelial cell and neutrophil viability,** measured by alamarBlue. TIGR4 THY data is replotted from Figure 1B and C as a comparison. Data are depicted as mean ± SEM with individual biological replicates shown. Mann-Whitney U-test; ns: not significant, * (P<0.05), ** (P<0.01), *** (P<0.001), **** (P<0.0001).

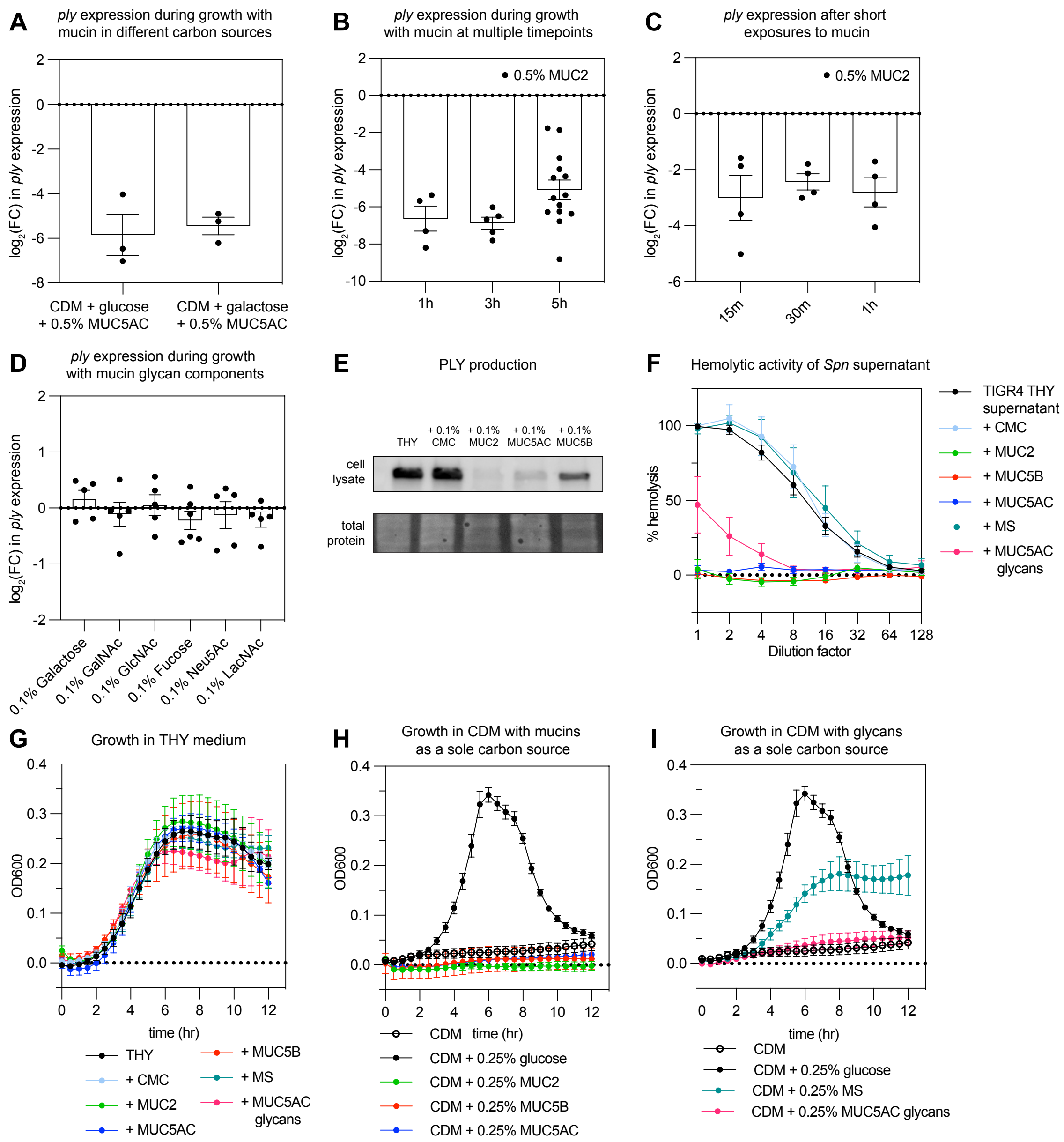

Figure S2. Mucins downregulate PLY over multiple growth phases without impacting growth. (A) *Ply* expression was measured by RT-qPCR after 5 h of growth in CDM supplemented with glucose or galactose. **(B)** *Ply* expression after 1, 3, or 5 h of growth with mucins. **(C)** *Ply* expression after 15 min, 30 min, and 1 h of exposure to mucin after 5 h of total growth. **(D)** *Ply* expression after 5 h of growth in THY supplemented with monosaccharides (MS) and disaccharides. **(A-D)** Bars show mean ± SEM with individual biological replicates shown. **(E)** Western blot analysis of PLY protein levels after mucin or CMC treatment in *Spn* cell lysates after 5 h. Total protein stain was used as a loading control. **(F)** Hemolytic activity of *Spn* culture supernatant (5 h) was assessed by absorbance of serially diluted supernatants at 540 nm relative to the WT and a PBS control. *Spn* was grown in THY medium supplemented with mucin, CMC, mucin glycans, or monosaccharides (MS). Data are the mean percentage of hemolysis ± SEM for n ≥ 3 biological replicates. **(G)** *Spn* growth in THY supplemented with mucins, mucin glycans, or a pool of monosaccharides (MS). **(H-I)** *Spn* growth in CDM with

mucins **(H)** or glycans **(I)**. **(I)** CDM and CDM + glucose data are replotted for comparison. **(G-I)** Data are the mean OD at 600 nm ± SEM for n ≥ 3 biological replicates.

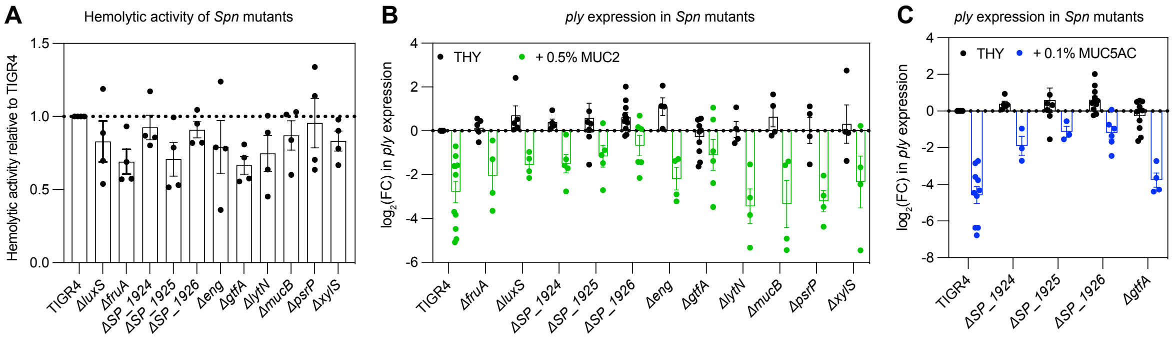

Figure S3. Putative regulators of *ply* do not contribute to mucin regulation. (A) Hemolytic activity of *Spn* mutants was assessed by absorbance of serially diluted 5-h supernatants at 540 nm relative to the WT and a PBS control. Data are the mean percentage of hemolysis ± SEM for n ≥ 3 biological replicates. **(B-C)** *Ply* expression in *Spn* mutants after 5 h of growth with and without MUC2 **(B)** or MUC5AC **(C)**. Bars show mean ± SEM with individual biological replicates shown.

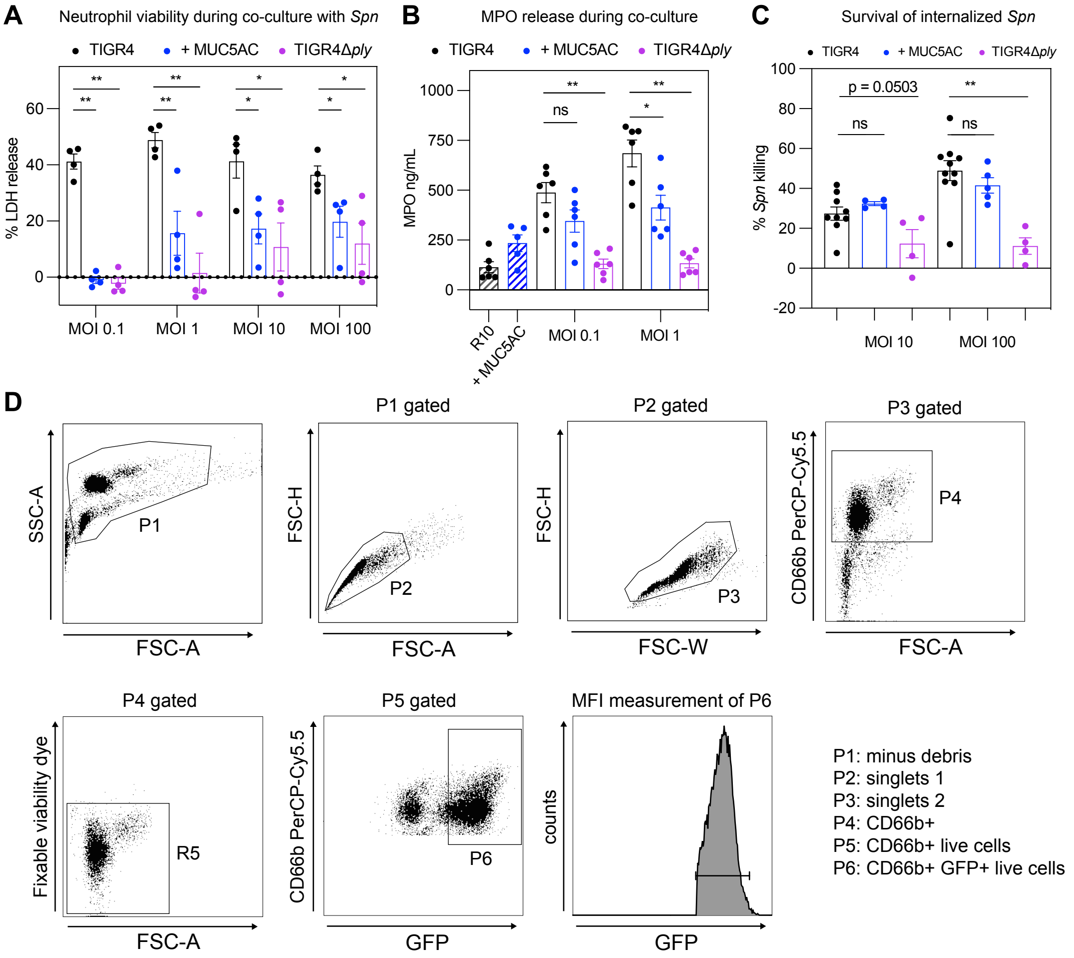

Figure S4. Mucins enhance neutrophil survival and modulate bactericidal activity. (A) Mucins enhance neutrophil survival during co-culture with live *Spn* TIGR4 over 16 h, as assayed by LDH release. **(B)** Mucins modulate the secretion of MPO upon *Spn* and neutrophil interaction, measured by ELISA. **(C)** Mucins do not alter bacterial killing efficiency, as inferred from the change in GFP MFI shown in **Fig. 1i** over 2 h. **(A-C)** Data are depicted as mean ± SEM with individual biological replicates shown. Mann-Whitney U-test; ns: not significant, * (P<0.05), ** (P<0.01), *** (P<0.001), **** (P<0.0001). **(D)** Dot plots and gating strategy used to identify neutrophils that have engulfed *Spn* (CD66+ GFP+) via flow cytometry. The MFI was analyzed for each sample. FSC: forward scatter; SSC: side scatter.

Methods

Strains and reagents

This study used the *Streptococcus pneumoniae* (*Spn*) strain TIGR4, generously provided by Marc Lipsitch from the Harvard T.H. Chan School of Public Health. This strain, originally isolated from an adult male patient's blood, exhibits high virulence in murine infection models. The TIGR4Δ*ply* (1) mutant was obtained from Justin Thornton (Mississippi State University), and strain JWV500 (2) that expresses GFP was a gift from Jan-Willem Veening (University of Lausanne). For routine growth and preservation, we cultured the bacteria on tryptic soy agar with 5% sheep blood (TSB agar) at 37°C in a 5% CO_2 atmosphere. Cultures for experiments were grown in Todd Hewitt broth supplemented with 0.5% yeast extract (THY broth) or chemically defined medium (CDM) (3) supplemented with 0.1% choline. When required, media were supplemented with erythromycin (0.5 µg/mL) or chloramphenicol (4.5 µg/mL) for antibiotic-resistant strains. Where indicated, we supplemented the medium with a pool of monosaccharides that comprise mucin glycans by combining equal amounts of galactose, *N*-acetylglucosamine, *N*-acetylgalactosamine, fucose and sialic acid. We monitored growth kinetics in THY broth with additives such as carboxymethyl cellulose (CMC) or mucin at 37°C with 5% CO_2, measuring the OD at 30-min intervals using a Synergy H1 microplate reader (BioTek).

Mutant strain construction

To generate TIGR4-*hlpA*-GFP and TIGR4Δ*ply-hlpA*-GFP strains, we amplified the GFP-tagged *hlpA* region from strain JWV500 using PCR with the Roche KAPA HiFi HotStart ReadyMix. This segment, carrying a chloramphenicol resistance cassette for selection, was then purified. To generate marked deletions of putative *ply* regulators, we amplified the up and downstream regions of the gene of interest followed by overlap extension PCR to insert the *ermB* gene. *Spn* was transformed with 100 ng of CSP-2 (AnaSpec) followed by the addition of the purified DNA. We selected mutants on TSB agar plates containing chloramphenicol or erythromycin.

Collection of human saliva

We collected submandibular saliva from healthy volunteers using a specialized vacuum pump system. Post-collection, the saliva was diluted with 5.5 M NaCl to achieve a final concentration of 0.16 M. To inhibit microbial growth and proteolytic degradation, we treated the saliva with a combination of antimicrobial agents and protease inhibitors: sodium azide (0.04 wt%), benzamidine HCl (5 mM), dibromoacetophenone (1 mM), phenylmethylsulfonyl fluoride (1 mM), EDTA (5 mM at pH 7), Triton-X-100 (TX100; 0.5%), and tri-n-butyl phosphate (15%). The treated saliva was then flash-frozen in liquid nitrogen and stored at -80°C. To remove TX100, we used SDR HyperD resin (Sartorius).

Native mucin purification

We isolated native porcine gastric mucins (MUC5AC), porcine intestinal mucins (MUC2), and human salivary mucins (MUC5B) following previously established methods (4). Briefly, mucus was harvested from pig stomachs and intestines, dissolved in a NaCl buffer with protease inhibitors and sodium azide, and clarified by centrifugation at low speed (8,000g for 30 min) followed by ultracentrifugation (190,000g for 1 h at 4°C with a Beckman 50.2 Ti rotor). We filtered out residual fat and particulates using Whatman #3 paper. Human submandibular saliva was collected and mucins purified as outlined earlier. Both salivary and mucus mucins were

fractionated by size-exclusion chromatography on Sepharose CL-2B columns, desalted, concentrated, and dissolved in water. To eliminate potential phage or viral contaminants, we treated MUC2 and MUC5AC preparations with 0.5% TX100 and 0.15% tri-N-butyl phosphate. We then removed TX100 using SDR HyperD resin (Sartorius). Next, the mucins were lyophilized, rehydrated by overnight shaking at 4°C in water or appropriate media, and checked for purity via mass spectrometry. Mass spectrometry analysis typically reveals that mucin extracts from porcine stomach predominantly contain MUC5AC, with minor components such as MUC2, MUC5B, MUC6, histones, actin, and albumin.

Isolation of mucin glycans from mucins
We dissociated non-reduced glycans from mucins using non-reductive alkaline β-elimination ammonolysis as previously detailed (4, 5). Commercial porcine gastric mucin (Sigma) was used for glycan isolation. Although commercially available mucin lacks structural and rheological properties compared to native mucins, isolated glycans from commercially available mucin are highly similar. Mucin was cleaned by ethanol precipitation, redissolved in PBS, and then desalted and lyophilized after ethanol removal. The mucins were treated with ammonium hydroxide and ammonium carbonate and incubated at 60°C for 50-60 h, resulting in glycosylamines and partially deglycosylated mucins. Volatile compounds were evaporated by centrifugation. Glycosylamines were separated from residual mucins via 10-kDa cut-off centrifugal filters (Amicon Ultracel), converted to oligosaccharide hemiacetals with boric acid, and purified with Hypercarb minicolumns (Thermo Fisher Scientific).

RNA preparation for gene expression analysis
We cultured *Spn* on TSB agar plates overnight. We then used the resulting colonies to start pre-cultures in THY broth. Once these cultures reached an OD of approximately 0.4, they were diluted to an OD of 0.01, equivalent to approximately 10^7 CFU/mL, in THY broth enriched with either 0.5% CMC, 0.5% mucin, 0.1% monosaccharides, or 0.1% mucin glycans. After incubation for 5 h at 37°C in a 5% CO_2 atmosphere, we harvested the bacteria at mid-log phase by centrifugation at 16,000g. The supernatant was discarded, and the bacterial pellets were flash-frozen in liquid nitrogen and stored at -80°C. For RNA extraction, the frozen pellets were thawed on ice, resuspended in 25 µL of lysozyme buffer, treated with Ready-Lyse Lysozyme (Biosearch Technologies) for 15 min at room temperature, and then processed via the MasterPure RNA Purification (Biosearch Technologies) kit. Any contaminating genomic DNA was eliminated with the Turbo DNA-free kit (Invitrogen), and the RNA samples were preserved at -20°C.

RNA sequencing analysis
For RNA sequencing analysis, we used three biological replicates per experimental condition. We prepared RNA libraries using the NEB RNA Ultra II kit with ribodepletion. We sequenced these libraries on the Illumina NextSeq platform, utilizing a single-end protocol with 92-nucleotide read lengths. We aligned the sequencing reads to the TIGR4 genome (NC_003028.3) via the Burrows–Wheeler aligner, with expression quantified using featureCounts on the Galaxy platform. We determined differential gene expression and statistical significance using the DESeq2 package within the R Bioconductor suite, applying the Benjamini-Hochberg procedure to adjust *P*-values.
To assess the biological implications of gene expression changes, we conducted gene ontology analysis to pinpoint biological processes overrepresented among the significantly regulated

genes. We conducted this analysis by using the BioCyc database, with Fisher's exact test evaluating the enrichment significance. We applied the Benjamini-Hochberg method to adjust *P*-values for multiple-hypothesis testing.

RT-qPCR analysis

We synthesized cDNA using the ProtoScript II First-Strand cDNA Synthesis kit (New England Biolabs). The elimination of genomic DNA was confirmed by qPCR amplification on negative control samples that did not have reverse transcriptase during cDNA synthesis. We used 8 ng of cDNA as a template for RT-qPCR with the SYBR Green Master Mix (Thermo Fisher Scientific), performed in a LightCycler 480 II real-time PCR instrument (Roche). We added forward and reverse primers at 3 μM each. Melting-curve analyses verified amplification of a single product. Gene expression changes were calculated on the basis of mean change in the qPCR cycle threshold compared with *gyrB* or *16S* (ΔC_t) and are reported as $\log_2[FC] = \Delta C_t\_CM - \Delta C_t\_sample$. Each sample was analyzed with at least three technical replicates.

Western blot

For western blot analysis, we processed *Spn* cell lysates and culture supernatants separately. For cell lysates, bacteria were cultured in THY broth with 0.1% CMC or mucin for 5 h, washed, flash-frozen, and stored at -80°C. Upon thawing, cells were lysed by using B-PER Reagent (Thermo Fisher Scientific) with the addition of Halt Protease Inhibitor Cocktail (Thermo Scientific). We determined protein concentrations with the Pierce BCA Protein Assay. For supernatant analysis, bacteria were incubated overnight in THY with 0.1% CMC or mucin. The supernatant was collected and treated with protease inhibitor. For blotting, 5 μg of lysate or 35 μL of supernatant was mixed with Laemmli buffer, heated, separated on a 10% TGX gel (Bio-Rad), and electro-transferred to nitrocellulose membranes. Post-transfer, we used No-Stain Protein Labeling Reagent (Invitrogen) for total protein visualization. Membranes were blocked in milk/PBS-Tween, incubated with an anti-PLY antibody (Abcam ab71811), washed, and then incubated with an HRP-conjugated secondary antibody. After a final wash, we applied the SuperSignal West Femto Maximum Sensitivity Substrate (Thermo Scientific) for band detection, visualized with a ChemiDoc imager (Bio-Rad).

Hemolysis assay

The amount of PLY released into the culture supernatant was determined using a hemolytic assay. We took supernatants from 5-h *Spn* cultures grown with and without mucin, mucin glycans, a pool of monosaccharides, or CMC, and performed serial dilutions in PBS with 0.1% dithiothreitol. To these dilutions, we added a 2% solution of sheep red blood cells in PBS and incubated the mixture at 37°C for 1 h. For controls, we used water for complete hemolysis (100%) and PBS for no hemolysis (0%). Post-incubation, the red blood cells were centrifuged down, the supernatant carefully removed, and its absorbance measured at 540 nm to quantify the extent of hemolysis.

Human cell culture

We used certified mycoplasma-free A549 cells (American Type Culture Collection [ATCC], CCL-184), a human lung epithelial carcinoma cell line. We regularly assessed the cells' morphology to ensure authenticity and conducted PCR tests to rule out contamination. The A549

cells were cultured in F-12K medium with 10% FBS at 37°C in a 5% CO_2 environment and passaged at a 1:12 ratio upon reaching confluency. For bacterial co-culture experiments, the cells were seeded in glass-bottom 96-well plates until confluent.
We isolated human neutrophils from blood obtained from healthy donors purchased from Research Blood Components. The blood was treated with HetaSep in a 1:5 ratio and incubated at 37°C for 30 min to separate leukocytes from erythrocytes. We then transferred the leukocyte-rich fraction to a new tube, lysed the erythrocytes with lysis buffer, and washed the cell mixture in PBS. Neutrophils were subsequently isolated using the EasySep Direct Human Neutrophil Isolation kit (Stemcell Technologies), following the provided protocol. We suspended the isolated neutrophils in either PBS or R10 medium for subsequent assays.

Human cell visualization

Spn was inoculated at 10^8 CFU/mL and grown for 3 h with or without mucin. Post-culture, we separated and purified the supernatant by passing it through 0.22-μm filters to eliminate any bacterial cells. We then applied this filtered supernatant, along with a THY medium control, in triplicate to A549 cell monolayers and neutrophils seeded in glass-bottom 96-well plates. The plates were incubated at 37°C with 5% CO_2 for 60 min. Subsequently, we added the LIVE/DEAD Cell Imaging kit (Invitrogen R37601), followed by incubation at room temperature for 15 min. We captured images using a Zeiss LSM 800 confocal microscope with a 63× oil immersion lens and analyzed the images with Zeiss ZEN software version 2.1. The images provided are representative of the observed results.

Human cell cytotoxicity assay

Spn was cultured at a concentration of 10^8 CFU/mL and incubated for 3 h with and without mucin. Following incubation, supernatants were separated and passed through 0.22-μm filters to remove bacterial cells. We added these filtered supernatants, along with THY medium controls, to A549 cells or neutrophils, which were suspended at 10^5 cells per well in a 96-well plate, set up in triplicate. To assess cell viability, we added alamarBlue HS cell viability reagent (Invitrogen A50100) and incubated the plates at 37°C with 5% CO_2. After 2 h, we measured fluorescence at 560/590 nm (excitation/emission) using a plate reader. Cell viability percentages were calculated relative to the medium control and adjusted for background fluorescence. In assays with live bacteria, we suspended 4×10^5 neutrophils in 400 μL of R10 medium with or without 0.1% MUC5AC. We then added *Spn* TIGR4 or TIGR4Δ*ply* at multiple MOIs (0.1, 1, 10, and 100) and incubated the mixtures overnight for 16 h at 37°C with 5% CO_2. The next day, we collected the supernatants and quantified the lactate dehydrogenase (LDH) release using an LDH release assay kit (Abcam a65393) as per the manufacturer's protocol.

ELISA

To assess cytokine production by neutrophils, we resuspended 4×10^5 cells in 400 μL of R10 medium, with or without 0.1% MUC5AC. We then introduced *Spn* TIGR4 or TIGR4Δ*ply* at MOIs of 1 and 0.1. After incubating the co-cultures overnight for 16 h at 37°C with 5% CO_2, we harvested the supernatants. We measured MPO and IL-1β concentrations in the supernatants using BioLegend LEGEND MAX ELISA kits according to the manufacturer's instructions.

Neutrophil viability and phagocytosis

To assess neutrophil phagocytosis and bactericidal activity, we prepared a suspension of 5×10^5 neutrophils in Hank's balanced salt solution fortified with calcium and magnesium (HBSS++; Thermo Fisher Scientific), with or without 0.1% MUC5AC. We treated GFP-tagged *Spn* TIGR4 and TIGR4Δ*ply* with 50% human serum in HBSS++ to opsonize the bacteria, then incubated this mixture at 37°C with 5% CO_2 for 30 min. The neutrophils were then exposed to the opsonized bacteria at MOIs of 10 and 100 and incubated for 1 h under the same conditions to allow phagocytosis. Following phagocytosis, we applied gentamicin at 100 µg/mL for 5 min at room temperature to eliminate any bacteria not internalized by the neutrophils. After two washes with PBS, the neutrophils were either stained immediately or underwent further incubation for 1 or 2 h, after which we analyzed the neutrophils and internalized GFP-positive *Spn* using flow cytometry.

Flow cytometry

For flow cytometry, we labeled neutrophils with CD66b-PerCP/Cy5.5 (BioLegend) at a 1:100 dilution and Fixable Viability Dye (eBioscience) at a 1:1,000 dilution in FACS buffer, a solution of PBS supplemented with 0.1% BSA. This staining process was carried out for 25 min at 4°C in the dark. Following staining, cells were washed in PBS, centrifuged, and fixed in a 1% formaldehyde solution in PBS overnight at 4°C. The next day, the samples were washed again in PBS, centrifuged, resuspended in 200 µL of FACS buffer, and filtered through a 35-µm strainer. We then analyzed the stained cells on a FACS Celesta flow cytometer (BD Biosciences). For data analysis, we used FlowJo software version 10.

***In vivo* intratracheal infection model**

Mice were housed in a specific pathogen-free facility with a 12-h light/dark cycle on pre-bedded corn cob disposable cages (Innovive). Mice received acidified water and 2020X chow (Envigo). For intratracheal infections, mice were anesthetized with 100 mg/kg ketamine and 10 mg/kg xylazine prior to visualization of the vocal cords via an otolaryngoscope (Welch Allyn). We infected 8- to 10-week-old C57BL/6 mice (Jackson Labs) with ~5×10^6 CFU TIGR4 WT grown in the presence of THY, 0.1% MUC5AC, or 0.1% MUC5AC glycans. At 8 h post infection, mice received intratracheal treatment of 40 µL THY, 0.1% MUC5AC, or 0.1% MUC5AC glycans after brief anesthesia with isoflurane. Mice were monitored for full recovery. At 24 h post infection, mice were humanely euthanized according to approved protocols. Blood was harvested by cardiac puncture, and lungs were homogenized. Blood and lungs were serially diluted and plated on TSB agar for enumeration.

Sex as a biological variable

Our study examined female mice. It is unknown whether the findings are relevant for male mice.

Statistics and reproducibility

All experiments were conducted with a minimum of three biological replicates. Data are reported as the mean ± SEM. Where applicable, we employed the Mann-Whitney U-test, the Wilcoxon signed-rank test, or the Kruskal-Wallis test with Dunn's correction for statistical analysis. The microscopy images provided are representative of consistent observations across multiple fields. We have verified that MUC2, MUC5AC, MUC5B, and their glycans show reproducible results across various purification batches.

Study approval

All animal studies were conducted in accordance with federal regulations set forth in the Animal Welfare Act. All experiments were performed based on protocols approved by the IACUC at UCSD (#S00227M) and recommendations by the *Guide for the Care and Use of Laboratory Animals* (6).

Data availability

The high-throughput sequencing data from this study will be accessible in the Gene Expression Omnibus database under accession number GSE265984. Values for all data points in graphs are reported in the Supporting Data Values file.

Acknowledgments

This research was supported by NIH National Institute of Allergy and Infectious Diseases award R21AI167289 (K.R.), NIH grant R01AI176554 (V.N.), DFG grant SFB1449-431232613, sub-project B02 (G.N., C.G.), National Science Foundation Microbiome Interactions and Mechanism grant EF-2125118 (K.R.), the US Army Research Office under cooperative agreement W911NF1920026 for the Institute for Collaborative Biotechnologies (K.R.), Toxicology Training Grant T32-ES007020 (J.B.), the A.P. Giannini Foundation Postdoctoral Fellowship (E.B.), and Army Research Office Multidisciplinary University Research Initiative award W911NF2210185 (K.R.). We acknowledge support from the MIT BioMicro Center and National Institute of Environmental Health Sciences grant P30ES002109. Figure S1a was created in BioRender.

References

1. Bryant JC, et al. Pyruvate oxidase of Streptococcus pneumoniae contributes to pneumolysin release. *BMC Microbiol*. 2016;16(1):1–12.
2. Kjos M, et al. Bright fluorescent Streptococcus pneumoniae for live-cell imaging of host-pathogen interactions. *J Bacteriol*. 2015;197(5):807–818.
3. Van De Rijn I, Kessler RE. Growth Characteristics of Group A Streptococci in a New Chemically Defined Medium. *Infect Immun*. 1980;27(2):444–448.
4. Wheeler KM, et al. Mucin glycans attenuate the virulence of Pseudomonas aeruginosa in infection. *Nat Microbiol*. 2019;4(12):2146–2154.
5. Takagi J, et al. Mucin O-glycans are natural inhibitors of Candida albicans pathogenicity. *Nat Chem Biol*. 2022;18(7):762–773.
6. National Research Council (US) Committee for the Update of the Guide for the Care and Use of Laboratory Animals. *Guide for the Care and Use of Laboratory Animals*. 8th ed. Washington (DC): National Academies Press (US); 2011.
