# Supplementary material for: Mucins protect against Streptococcus pneumoniae virulence by suppressing pneumolysin expression
Source: J Clin Invest. 2024 Aug 22;134(19):e182769. doi: 10.1172/JCI182769 (PMC11444151; doi:10.1172/JCI182769)
Supplement: Unedited blot and gel images [file jci-134-182769-s038.pdf]

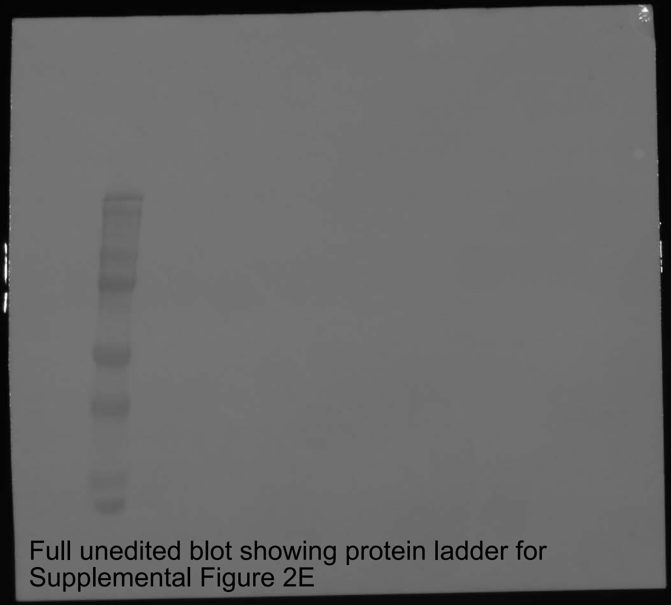

Full unedited blot showing protein ladder for
Supplemental Figure 2E

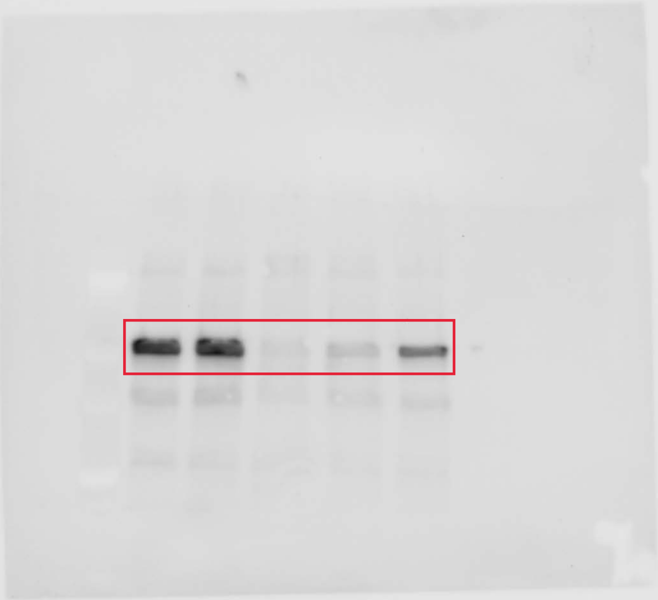

Full unedited blot for Supplemental Figure 2E

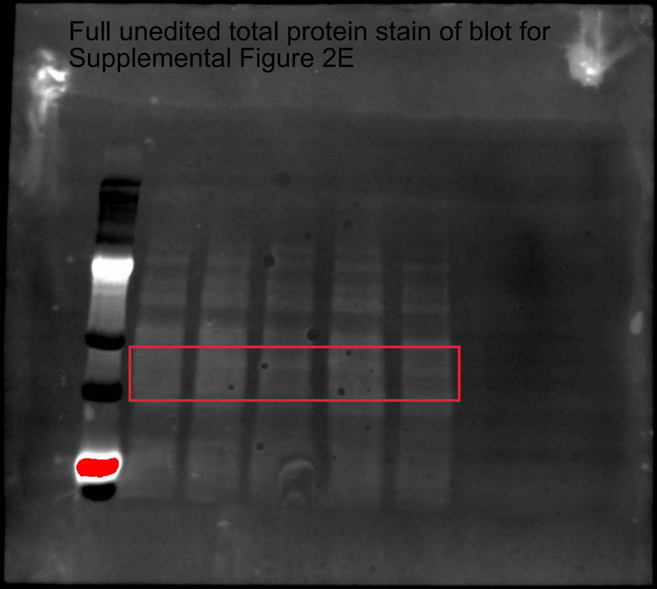
Full unedited total protein stain of blot for
Supplemental Figure 2E
